# Development and Validation of a Multivariable Lung Cancer Risk Prediction Model That Includes Low-Dose Computed Tomography Screening Results: A Secondary Analysis of Data From the National Lung Screening Trial

**DOI:** 10.1001/jamanetworkopen.2019.0204

**Published:** 2019-03-01

**Authors:** Martin C. Tammemägi, Kevin ten Haaf, Iakovos Toumazis, Chung Yin Kong, Summer S. Han, Jihyoun Jeon, John Commins, Thomas Riley, Rafael Meza

**Affiliations:** 1 Department of Health Sciences, Brock University, St Catharines, Ontario, Canada; 2 Department of Public Health, Erasmus MC University Medical Center, Rotterdam, the Netherlands; 3 Department of Radiology, Stanford University School of Medicine, Palo Alto, California; 4 Institute for Technology Assessment, Massachusetts General Hospital, Harvard Medical School, Boston; 5 Department of Medicine, Stanford University School of Medicine, Palo Alto, California; 6 School of Public Health, University of Michigan, Ann Arbor; 7 Information Management Systems, Rockville, Maryland; 8 Department of Epidemiology, University of Michigan, Ann Arbor

## Abstract

**Importance:**

Low-dose computed tomography lung cancer screening is most effective when applied to high-risk individuals.

**Objectives:**

To develop and validate a risk prediction model that incorporates low-dose computed tomography screening results.

**Design, Setting, and Participants:**

A logistic regression risk model was developed in National Lung Screening Trial (NLST) Lung Screening Study (LSS) data and was validated in NLST American College of Radiology Imaging Network (ACRIN) data. The NLST was a randomized clinical trial that recruited participants between August 2002 and April 2004, with follow-up to December 31, 2009. This secondary analysis of data from the NLST took place between August 10, 2013, and November 1, 2018. Included were LSS (n = 14 576) and ACRIN (n = 7653) participants who had 3 screens, adequate follow-up, and complete predictor information.

**Main Outcomes and Measures:**

Incident lung cancers occurring 1 to 4 years after the third screen (202 LSS and 96 ACRIN). Predictors included scores from the validated PLCOm2012 risk model and Lung CT Screening Reporting & Data System (Lung-RADS) screening results.

**Results:**

Overall, the mean (SD) age of 22 229 participants was 61.3 (5.0) years, 59.3% were male, and 90.9% were of non-Hispanic white race/ethnicity. During follow-up, 298 lung cancers were diagnosed in 22 229 individuals (1.3%). Eight result combinations were pooled into 4 groups based on similar associations. Adjusted for PLCOm2012 risks, compared with participants with 3 negative screens, participants with 1 positive screen and last negative had an odds ratio (OR) of 1.93 (95% CI, 1.34-2.76), and participants with 2 positive screens with last negative or 2 negative screens with last positive had an OR of 2.66 (95% CI, 1.60-4.43); when 2 or more screens were positive with last positive, the OR was 8.97 (95% CI, 5.76-13.97). In ACRIN validation data, the model that included PLCOm2012 scores and screening results (PLCO2012results) demonstrated significantly greater discrimination (area under the curve, 0.761; 95% CI, 0.716-0.799) than when screening results were excluded (PLCOm2012) (area under the curve, 0.687; 95% CI, 0.645-0.728) (*P* < .001). In ACRIN validation data, PLCO2012results demonstrated good calibration. Individuals who had initial negative scans but elevated PLCOm2012 six-year risks of at least 2.6% did not have risks decline below the 1.5% screening eligibility criterion when subsequent screens were negative.

**Conclusions and Relevance:**

According to this analysis, some individuals with elevated risk scores who have negative initial screens remain at elevated risks, warranting annual screening. Positive screens seem to increase baseline risk scores and may identify high-risk individuals for continued screening and enrollment into clinical trials.

**Trial Registration:**

ClinicalTrials.gov Identifier: NCT00047385

## Key Points

**Question:**

In this study of data from the National Lung Screening Trial (NLST), can a lung cancer risk model’s prediction be improved by inclusion of lung cancer screening results?

**Findings:**

In this secondary analysis of NLST data including 22 229 participants, a model incorporating a validated lung cancer risk prediction model, the PLCOm2012 model, with National Lung Screening Trial results (PLCO2012results) predicted future lung cancer significantly better than a model excluding results.

**Meaning:**

The PLCO2012results model estimates may improve stratification of patients being screened for lung cancer into high- and low-risk strata and may help guide decision making regarding screening interval.

## Teaser

This secondary analysis of data from the National Lung Screening Trial develops and validates a risk prediction model that incorporates low-dose computed tomography (CT) screening results for patients being screened for lung cancer.

## Introduction

Lung cancer is the leading cause of cancer death in the world.^1^ The National Lung Screening Trial (NLST)^2^ demonstrated a 20% lung cancer mortality reduction for low-dose computed tomography (LDCT) screening of high-risk individuals compared with chest radiography screening. Consequently, the US Preventive Services Task Force^3^ and the Centers for Medicare & Medicaid Services^4^ have recommended annual screening for high-risk individuals, including current and former smokers, who quit less than 15 full years ago, smoked at least 30 pack-years, and are aged 55 to 80 years or 55 to 77 years, respectively. These criteria are identical to NLST enrollment criteria, except for increasing the upper age limit beyond 74 years. Lung cancer screening programs using these criteria are being implemented across the United States. Major concerns regarding lung cancer screening are the harms and costs of the high number of false-positive screens and excess radiation exposure.^5^ Eliminating nonbeneficial screens is expected to reduce the harms and improve cost-effectiveness. It has been demonstrated that, compared with NLST-like criteria, accurate lung cancer risk prediction models are more sensitive in selecting individuals who develop lung cancer, have higher positive predictive values, have a lower number needed to screen to avert 1 lung cancer death, and are more cost-effective.^6,7,8,9,10^ The PLCOm2012 is a lung cancer risk prediction model that has been validated by research teams in several countries, including the United States, Germany, Australia, and Canada.^6,11,12,13,14,15,16^ One appropriate PLCOm2012 risk threshold for identifying individuals for screening is at least 1.5% 6-year risk.^8^

Evidence suggests that lung cancer screening results predict future lung cancer risk. The Dutch-Belgian Lung Cancer Screening (NELSON) trial demonstrated that the results of the initial screen were associated with subsequent incidence of lung cancer in 5.5 years of follow-up.^17^ The results of the first 3 screenings, especially the third screening round, were indicative of the risk of lung cancer detected in the fourth screening round, which occurred 2.5 years later. Data from the Continuous Observation of Smoking Subject (COSMOS) trial^18^ were used to develop a model that included nodule features from the initial screen that predicted lung cancer identified at subsequent screens. In the NLST,^19^ individuals with a negative initial screen result had lower subsequent lung cancer risks. We hypothesized that prediction of future lung cancer risk could be improved by combining information from screening results with risk prediction model estimates. Such updated risk estimates could help determine whether one might defer the next annual screen owing to low risk or more strongly recommend the next screen owing to high risk.

The American College of Radiology developed the Lung CT Screening Reporting & Data System (Lung-RADS),^20^ which has become commonly used in screening practice in North America. Lung-RADS 1 (no nodule or nonsuspicious nodule) and Lung-RADS 2 (nodule present with benign appearance or behavior, having very low likelihood of becoming a clinically active cancer) are described as negative in that they do not trigger additional surveillance imaging or clinical diagnostic investigation. Lung-RADS 3 indicates the presence of a probably benign nodule, which has a low likelihood of becoming a clinically active cancer, and a 6-month surveillance scan is recommended. Lung-RADS 4A, 4B, and 4X indicate a screening result that is suspicious for lung cancer, which requires further investigation that can range from a 3-month surveillance scan to tissue biopsy. In the present study, we use NLST screening results classified into dichotomized Lung-RADS screening results as positive (Lung-RADS 3 or 4) vs negative (Lung-RADS 1 or 2). In NLST data, we evaluate the association of PLCOm2012 risk and 3 annual screening results with lung cancer risk (incidence) from 1 to 4 years after the last screen, and we develop and validate a lung cancer risk prediction model that describes these associations. Of interest was the association of screening results with future lung cancer risk to help guide future screening decision making.

## Methods

### Study Design

The NLST (ClinicalTrials.gov identifier NCT00047385) design has been described.^2,21^ Briefly, the NLST was a randomized clinical screening trial comparing the effect of LDCT vs chest radiography screening on lung cancer mortality. Between August 2002 and April 2004, a total of 53 452 participants were enrolled and scheduled to receive screens at baseline (T0), 1 year (T1), and 2 years (T2). Participants were followed up until December 31, 2009. The present prediction modeling analysis took place between August 10, 2013, and November 1, 2018. Epidemiological data were collected at study entry by structured questionnaires. The NLST institutional review board approval was obtained at each participating center and the National Cancer Institute (NCI), and informed written consent was obtained from all participants. This study followed the Transparent Reporting of a Multivariable Prediction Model for Individual Prognosis or Diagnosis (TRIPOD)^33^ reporting guidelines.

The NLST was a collaborative effort, including the Lung Screening Study (LSS) component (10 sites administered by an NCI Division of Cancer Prevention contract) and the American College of Radiology Imaging Network (ACRIN) component (23 sites administered through an NCI Division of Cancer Treatment and Diagnosis grant). The LSS sites enrolled 34 612 participants (64.8%), and ACRIN enrolled 18 840 participants (35.2%). An Annual Study Update (ASU), a self-reported questionnaire, updated participant information on smoking each year. The ACRIN and LSS components of the NLST collected ASU data differently, and we carried out a sensitivity analysis using LSS data only as part of this study.

In the present secondary analysis of data from the NLST, screening results are based retrospectively on the Lung-RADS definition.^20^ A comparison of the NLST criteria and the Lung-RADS classification for a positive screen, which considers Lung-RADS 2 to be negative, in NLST data has been published,^22^ and we use identical coding. The present analysis included individuals who completed all 3 LDCT screens and were lung cancer free 1 year after the third screen (23 574 of 26 722 [88.2%]) (eFigure 1 in the Supplement).

### Statistical Analysis

The present study evaluates the association of lung cancer screening results with subsequent lung cancer risk, adjusted for other important lung cancer risk predictors. The latter were estimated jointly by risks estimated using the PLCOm2012 model.^6,8^ Complete-cases multivariable fractional polynomial logistic regression models^23^ were used to predict lung cancers occurring 1 to 4 years after the last screen (T3 to T6) using PLCOm2012-estimated risks with and without screening results as predictors. Eight combinations of the dichotomous Lung-RADS screening results in the 3 annual screens were evaluated as predictors (described later in the Screening Findings and Prediction Models subsection of the Results section). Lung cancers occurring during the NLST screening period (T0 to T3) were excluded because we were interested in studying the additional information screening results provide on subsequent lung cancer risk beyond 3 screens, not whether a specific abnormal screen represented lung cancer. Also, lung cancers detected more than 1 year after the last screen were primarily clinically detected and avoid screening-associated overdiagnosis.

**Figure 1.**
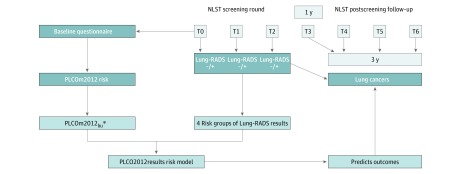
Schema Showing PLCO2012results Model Development Plan Lung-RADS indicates Lung CT Screening Reporting & Data System developed by the American College of Radiology; NLST, National Lung Screening Trial; PLCOm2012, risk prediction model described by Tammemägi et al^6^; PLCOm2012_bu_, PLCOm2012 model with predictors age, smoking duration in current smokers, and quit time in former smokers updated to the start of study follow-up (T3) by adding 3 years to baseline values (the PLCOm2012_bu_ is estimated for a 3-year period, not the original 6-year period); T0, baseline screen; T1, first annual screen; and T2, annual screen at year 2.

Limited follow-up remained between the start of T3 and the trial’s completion. Most individuals did not have 6 years of follow-up from the start of T3, as is used by PLCOm2012, but did have 3.5 years of follow-up. For our study, follow-up was truncated at 6 years since randomization, providing 3 years of follow-up. A schema demonstrating the study design used for model preparation is shown in Figure 1. Cumulative incidences of lung cancer from T3 to T6 were estimated, stratified by screening results, taking competing risks into account.^24^

Selected PLCOm2012 predictor variable data were updated from baseline to T3, this study’s follow-up onset. Age, smoking duration in baseline current smokers, and smoking quit time in baseline former smokers were updated to T3 by adding 3 years to the T0 baseline values. The PLCOm2012-estimated risks were converted from 6-year risk to 3-year risk by dividing by 2 to match this study’s follow-up period. The label PLCOm2012_bu_ identifies these baseline-updated 3-year PLCOm2012 risks. This updating potentially introduces some error in smoking duration and quit time at T3 because some baseline current smokers may have stopped smoking by then and some baseline former smokers may have relapsed. The NLST LSS ASU information^25^ indicates that 24.1% of baseline current smokers had quit by T3; in the Prostate, Lung, Colorectal and Ovarian Cancer Screening Trial,^26^ smoking relapse occurred in 3.3% of baseline former smokers after 8.5 years of follow-up. In LSS data, a subset analysis was conducted updating smoking information using ASU information. These ASU-updated data did not improve prediction and are thus not used in the present analysis for consistency across the LSS and ACRIN data sets.

The LSS data were used to develop a PLCOm2012_bu_-based model incorporating screening results, and ACRIN data were used to validate the model. The ACRIN data were used for validation instead of a random sampling of the entire NLST LDCT group because they replicate a true external validation sample having different geographies, populations, practices, and radiologists.

Overall model prediction was assessed using the Brier score (zero indicates perfect prediction, and 0.25 indicates random prediction).^27^ Discrimination, the ability to correctly classify individuals who develop lung cancer, was assessed using the receiver operating characteristic area under the curve (AUC). Calibration, how closely predicted probabilities match observed probabilities, was assessed by estimating the 50th and 90th percentiles of absolute error (difference between the observed and predicted).^28^ Calibration was also evaluated by plotting the observed vs predicted risks and by evaluating the *P* value for the Spiegelhalter statistic (significance indicates some inconsistency). For Brier scores and AUCs, 95% CIs were estimated using bias-corrected percentile intervals in 1000 bootstrap resamplings.

Decision curve analysis (DCA) comparing the net benefit of PLCOm2012_bu_ plus screening results vs PLCOm2012_bu_ only was carried out.^29^ This is described in the eAppendix in the Supplement.

Regarding descriptive statistics, for tests of proportions and rates, 95% CIs and *P* values were prepared using methods described by Brown and colleagues^30^ and by Miettinen.^31^ Testing differences in continuous variables with normal distributions used *t* test, not assuming equal variances, in skewed continuous variables used nonparametric test of trend,^32^ and for proportions used the χ^2^ test or Fisher exact test.

Statistical software (Stata 15.1 MP; StataCorp LP) was used for the analyses. Hypothesis testing used 2-sided *P* values, and an α error less than .05 determined statistical significance.

## Results

Overall, 22 229 individuals (14 576 LSS and 7653 ACRIN) were studied. The mean (SD) age of participants was 61.3 (5.0) years, 59.3% were male, and 90.9% were of non-Hispanic white race/ethnicity. eFigure 1 and eTable 1 in the Supplement summarize study participant selection and characteristics, respectively. Overall, during follow-up from T3 to T6, a total of 298 lung cancers (202 LSS and 96 ACRIN) were diagnosed in the 22 229 individuals (1.3%) (14 576 in LSS and 7653 in ACRIN) with complete data for modeling. With a total cumulative incidence of 0.0134 (95% CI, 0.0119-0.0150) and with 64 921 person-years of follow-up, the incidence rate was 459 (95% CI, 408-514) per 100 000 person-years, including 474 (95% CI, 410-544) in LSS and 431 (95% CI, 349-526) in ACRIN (*P* = .45).

### Screening Findings and Prediction Models

**Figure 2.**
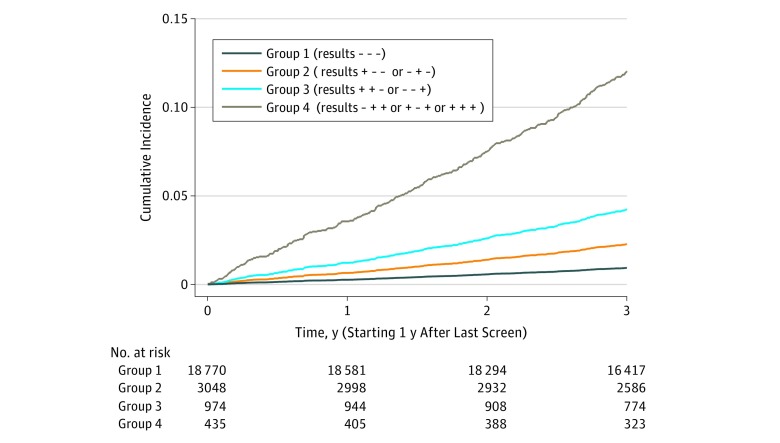
Cumulative Incidence of Lung Cancer in the National Lung Screening Trial Low-Dose Computed Tomography Group Occurring 1 to 4 Years After the Last Low-Dose Computed Tomography Screen Among 23 227 Participants^a^ Stratified by Lung CT Screening Reporting & Data System screen results (positive vs negative) at baseline, 1-year, and 2-year annual screenings categorized into 4 groups. ^a^Competing risks (ie, non–lung cancer deaths) were taken into account according to the method by Fine and Gray.^24^ Included in the analysis were 298 incident lung cancer cases and 735 competing-cause deaths during the 3-year follow-up period.

In LSS data, univariable and multivariable logistic regression models were developed predicting lung cancer 1 to 4 years after the last screen using Lung-RADS results with and without adjustment for PLCOm2012_bu_ scores as predictors (Table 1). These models were prepared using Lung-RADS results in 8 groupings of dichotomous screen results over the 3 annual screens. In addition, based on odds ratio magnitudes and reasoning regarding the associations of multiple vs single positive results and number of individuals per result group, the screening Lung-RADS results were categorized into the following 4 groups (Table 1): (1) all 3 screens negative (− − −), (2) 1 positive screen and last negative (+ − − or − + −), (3) 2 positive screens with last negative or 2 negative screens with last positive (+ + − or − − +), and (4) at least 2 positive screens with last positive (+ − + or − + + or + + +). Adjusted for PLCOm2012 risks, compared with participants with 3 negative screens, participants with 1 positive screen and last negative had an odds ratio (OR) of 1.93 (95% CI, 1.34-2.76), and participants with 2 positive screens with last negative or 2 negative screens with last positive had an OR of 2.66 (95% CI, 1.60-4.43); when 2 or more screens were positive with last positive, the OR was 8.97 (95% CI, 5.76-13.97) (Table 1). The distributions of screening results classified in the 4 groups in LSS and ACRIN are listed in eTable 2 in the Supplement. The distributions of screening results and lung cancer rates were similar in LSS and ACRIN. As the screening results group increases in cumulative risk of lung cancer (0.9%, 2.0%, 3.6%, and 10.1% for groups 1 through 4 overall, respectively), the frequency of occurrence of each results group declined in LSS, ACRIN, and overall (Figure 2 and eTable 2 in the Supplement). Generally, lung cancer risk increased with the number and recency of positive screens.

**Table 1.** Univariable and Multivariable Logistic Regression Model Odds Ratios for Predicting Lung Cancer Occurring 1 to 4 Years After the Third Screen, Stratified by 8-Level and 4-Level Screening Results, Prepared in LSS Data and Validated in ACRIN Data

Model Predictor^a^ or Predictive Performance Characteristic^b^	Lung Cancer, No./Total No. (Row %) [Column %] in Strata (n = 15 152)	Univariable Odds Ratio (95% CI) (n = 15 152)	*P* Value	Multivariable Odds Ratio (95% CI) [β Coefficient] (n = 14 576)^c^	*P* Value
**Screening Results in 8 Levels in LSS**					
− − −	116/12 223 (0.9) [80.7]	1 [Reference]	NA	1 [Reference]	NA
+ − −	32/1565 (2.0) [10.3]	2.17 (1.47-3.23)	<.001	1.86 (1.24-2.78)	.003
− + −	11/463 (2.4) [3.1]	2.54 (1.36-4.75)	.003	2.16 (1.15-4.05)	.02
+ + −	2/125 (1.6) [0.8]	1.70 (0.41-6.94)	.46	1.29 (0.31-5.30)	.73
− − +	16/496 (3.2) [3.3]	3.48 (2.05-5.91)	<.001	3.08 (1.80-5.25)	<.001
+ − +	15/147 (10.2) [1.0]	11.86 (6.74-20.86)	<.001	7.73 (4.11-14.53)	<.001
− + +	7/91 (7.7) [0.6]	8.70 (3.94-19.21)	<.001	6.50 (2.90-14.54)	<.001
+ + +	9/42 (21.4) [0.3]	28.46 (13.32-60.83)	<.001	19.03 (8.59-42.15)	<.001
PLCOm2012_bu_ risk	NA	Excluded	NA	Included, nonlinear^d^	<.001
**Predictive Performance in LSS**					
Brier score (95% CI)	NA	0.013 (0.012-0.015)	NA	0.013 (0.012-0.015)	NA
ROC AUC (95% CI)	NA	0.639 (0.601-0.676)	NA	0.772 (0.743-0.799)	NA
Spiegelhalter *P* value	NA	.50	NA	.47	NA
Mean probability O/E	NA	0.0137/0.0137	NA	0.0139/0.0139	NA
**Screening Results in 4 Levels in LSS**				**PLCO2012results Model**	
− − −	116/12 223 (0.9) [80.7]	1 [Reference]	NA	1 [Reference]	NA
+ − − or − + −	43/2028 (2.1) [13.4]	2.26 (1.59-3.20)	<.001	1.93 (1.34-2.76) [0.6554117]	<.001
+ + − or − − +	18/621 (2.9) [4.1]	3.12 (1.88-5.15)	<.001	2.66 (1.60-4.43) [0.9798233]	<.001
+ − + or − + + or + + +	31/280 (11.1) [1.8]	12.99 (8.57-19.69)	<.001	8.97 (5.76-13.97) [2.1940610]	<.001
PLCOm2012_bu_ risk score	NA	Excluded	NA	Nonlinear [−0.2713125]^d^	<.001
Model constant	NA	NA	NA	[−4.4353800]	NA
**Predictive Performance in LSS**					
Brier score (95% CI)	NA	0.013 (0.012-0.015)	NA	0.013 (0.012-0.015)	NA
ROC AUC (95% CI)	NA	0.638 (0.602-0.677)	NA	0.769 (0.741-0.797)	NA
Spiegelhalter *P* value	NA	.50	NA	.47	NA
Mean probability O/E	NA	NA	NA	0.0139/0.0139	NA
50th, 90th percentile absolute error	NA	NA	NA	0.0009, 0.0012	NA
**Predictive Performance of PLCO2012results in ACRIN (n = 7653)**					
Brier score (95% CI)	NA	NA	NA	0.012 (0.010-0.014)	NA
ROC AUC (95% CI)	NA	NA	NA	0.761 (0.716-0.799)	NA
Spiegelhalter *P* value	NA	NA	NA	.95	NA
Mean probability O/E	NA	NA	NA	0.0125/0.0149	NA
50th, 90th percentile absolute error	NA	NA	NA	0.0018, 0.0030	NA

Abbreviations: ACRIN, American College of Radiology Imaging Network subset of the National Lung Screening Trial; AUC, area under the curve; LSS, Lung Screening Study subset of the National Lung Screening Trial; NA, not applicable; O/E, observed/expected; ROC, receiver operating characteristic curve.

^a^ PLCOm2012 is a risk prediction model described by Tammemägi et al^6^; PLCOm2012_bu_, PLCOm2012 model with predictors age, smoking duration in current smokers, and quit time in former smokers updated to the start of study follow-up (T3) by adding 3 years to baseline values (the PLCOm2012_bu_ is estimated for a 3-year period, not the original 6-year period).

^b^ The minus sign represents a negative screen, and the plus sign represents a positive or abnormal screen suspicious for lung cancer. The results are presented in order for T0, T1, and T2 screens.

^c^ The multivariable model included adjusted for PLCOm2012_bu_ risks with predictors age, smoking duration in current smokers, and quit time in former smokers updated to 1 year after the last screen by adding 3 years to baseline values, and risk is estimated for 3-year follow-up.

^d^ PLCOm2012_bu_ is nonlinear and is transformed as follows: (PLCOm2012 base 3 year ^−0.5) − 7.045149954.

Considering first screens only, of the 22 167 NLST LDCT participants who had a Lung-RADS negative first screen, 264 (1.2%) had lung cancer diagnosed between T3 and T6. Of the 3156 individuals who had a positive first screen, 109 (3.5%) were diagnosed as having lung cancer in the same period, regardless of subsequent screening results.

In LSS, the PLCOm2012_bu_ risk-adjusted model with Lung-RADS results in 8 groupings had similar predictive performance as the model with results in 4 categories (Table 1). Overall prediction measured by the Brier score indicated good prediction for both models (0.013; 95% CI, 0.012-0.015 for both). Discrimination, measured by the AUC, was good for both models. For the adjusted 8-category risk model, the AUC was 0.772 (95% CI, 0.743-0.799); for the 4-category model, the AUC was 0.769 (95% CI, 0.741-0.797) (*P* = .07). The nonsignificant *P* values for Spiegelhalter statistics indicated good calibration for both models. In subsequent analyses, we used screening results in the 4 groupings and refer to the model that included PLCOm2012_bu_ and 4-group Lung-RADS results as the PLCO2012results model.

In ACRIN validation data, the PLCO2012results model had high overall prediction (Brier score, 0.012; 95% CI, 0.010-0.014), high discrimination (AUC, 0.761; 95% CI, 0.716-0.799), and good calibration as indicated by the nonsignificant Spiegelhalter statistic, as well as small 50th and 90th percentiles of absolute error (0.0018 and 0.0030, respectively). The observed and predicted mean probabilities were 1.25% and 1.49%, indicating that in the validation data the PLCO2012results model overestimates risk. This may in part be due to the ACRIN participants having a lower lung cancer incidence rate than LSS participants, in whom the model was developed. The plot of the observed and predicted probabilities (eFigure 2 in the Supplement) shows that the overestimation primarily occured above the 12.0% risk estimate, which is well above the range of risk thresholds considered for selecting individuals for lung cancer screening.^8,34^ Only 0.9% of the NLST LDCT sample had PLCO2012results risks exceeding 12.0%.

In ACRIN validation data, compared with the PLCOm2012_bu_ model, which excludes screening results, the PLCO2012results model demonstrated superior overall prediction (Brier score, 0.012 vs 0.013), discrimination (AUC, 0.761; 95% CI, 0.716-0.799 vs 0.687; 95% CI, 0.645-0.728) (*P* < .001) (eFigure 3 in the Supplement), and calibration (observed vs predicted, 0.84 vs 0.57). This analysis was also carried out applying the NLST criteria for positivity (≥4-mm nodules), and results were similar.

The sensitivity, specificity, and positive predictive values of the PLCOm2012_bu_ and PLCO2012results models for identifying lung cancers 1 to 4 years after the last screen using 0.8%, 1.0%, and 1.5% 3-year risk thresholds, stratified by LSS and ACRIN groups, are listed in eTable 3 in the Supplement. Those values indicate that the calibration of PLCOm2012_bu_ and PLCO2012results differed. For example, the sensitivities, specificities, and positive predictive values for PLCO2012results with a threshold of 1.0% were similar to those of PLCOm2012_bu_ with a threshold of 1.5% (85.4%, 48.9%, and 2.1% vs 84.4%, 47.2%, and 2.0%, respectively.

Decision curve analysis demonstrated that between risk thresholds 0% to 6.0% the net benefit for model PLCO2012results exceeded that of PLCOm2012_bu_ (eAppendix and eFigure 4 in the Supplement). In total, 99.1% of the overall NLST LDCT sample had PLCO2012results 3-year risks of 6.0% or less.

## Discussion

### Application of the PLCO2012results Model

Lung cancer screening results may predict future lung cancer risk. The PLCO2012results model can provide information to help guide decision making regarding whether to undertake or omit the next annual screen. An easy-to-use spreadsheet calculator that estimates PLCO2012results risks for individuals with recent lung cancer screening results is available online (https://brocku.ca/lung-cancer-risk-calculator). For a given risk threshold used to select individuals for lung cancer screening, applying the PLCO2012results can lead to reassignment of PLCOm2012 selection classification.

Having at least 1 positive screen was associated with increased PLCOm2012 risk. So, individuals who entered into the screening program based on a PLCOm2012 risk threshold would continue to have PLCO2012results model risks above the threshold and should continue with annual screening.

Having consecutive negative screens was associated with a lower lung cancer risk than indicated by the PLCOm2012 model, which does not account for screening results. Eligible individuals who were close to the screening threshold at the first screen and had consecutive negative screening results may have had a lower postscreening estimated risk than the eligibility threshold and could omit screening for 1 year. In contrast, there is a PLCOm2012 ceiling risk level at or above which the PLCOm2012 risk could not drop below a selected screening eligibility threshold even after 1, 2, or 3 consecutive negative screens. Individuals at or above the ceiling probability should continue annual screening. For example, if the eligibility threshold is 1.5%, individuals with PLCOm2012 risks of at least 2.6% should continue to receive annual screens because negative screens cannot lower 6-year risks below 1.5%. If the PLCOm2012 enrollment threshold is 2.00%, individuals with PLCOm2012 risks of at least 3.4% should continue screening independent of the negative results. Individual assessments would be required for those with risks above the threshold but below the ceiling and who have had negative screens.

In NLST, 92.1% of individuals with an initial negative screen had all 3 screens negative. Individuals with an initial negative screen who have PLCOm2012 risks below the ceiling probability for 1.5% or 2.0% enrollment thresholds have low 3-year lung cancer risks of 0.3% and 0.5%, respectively (Table 2), which might justify delaying the next screen by 1 year. In contrast, those individuals with initial negative screens who have PLCOm2012 risks above the ceiling probabilities are at high 3-year risks, 1.8% and 1.9%, warranting continued annual screening. With a 1.5% 6-year eligibility risk threshold, 56.0% of individuals with an initial negative screen should continue with annual screening because their baseline risks were at or above the ceiling threshold. Similarly, with a 2.0% threshold, 42.1% should continue with annual screening. Table 3 summarizes potential implications of the study findings of PLCOm2012 and PLCO2012results model on screening schedules.

**Table 2.** Distributions of Lung Cancer Outcomes Occurring 1 to 4 Years After the Last Screen in National Lung Screening Trial Participants^a^

Eligibility Threshold and Ceiling Probability^b^	No. (Row %)		Total, No. (Column %)
	No Lung Cancer	Lung Cancer	
**Eligibility Threshold 1.5% 6-y Risk**			
Probability <2.6%	8585 (99.7)	26 (0.3)	8611 (44.0)
Probability ≥2.6%	10 746 (98.2)	192 (1.8)	10 938 (56.0)
Total	19 331	218	19 549
**Eligibility Threshold 2.0% 6-y Risk**			
Probability <3.4%	11 254 (99.5)	58 (0.5)	11 312 (57.9)
Probability ≥3.4%	8077 (98.1)	160 (1.9)	8237 (42.1)
Total	19 331	218	19 549

^a^ Participants with an initial negative screen were stratified by PLCOm2012 (a risk prediction model described by Tammemägi et al^6^) ceiling probabilities for a 1.5% 6-year eligibility threshold (ceiling ≥2.6%) and 2.0% eligibility threshold (ceiling ≥3.4%). At the ceiling probability or above, having 3 consecutive negative screens will not lower the results-adjusted PLCOm2012 risk score below the eligibility threshold according to estimates prepared by the PLCO2012results model.

^b^ The probability is for 6-year risk estimated by PLCOm2012.

**Table 3.** Potential Implications of the Study Findings Regarding Offering the Next Annual Screen Based on PLCOm2012 and PLCO2012results Models

Criteria for Screening Selection	Lung-RADS Screening Result^a^			Idea
	First	Second	Third	
Risk prediction model threshold	− −	NA −	NA NA	Offer the next annual screen if PLCOm2012 ceiling probability is reached or exceeded for the chosen eligibility threshold. If PLCOm2012 risk is below the ceiling threshold, consider omitting the next annual screening. Based on NLST data, the probability of having 3 consecutive negative screens when an initial screen is negative is 92.1%. After an initial negative screen, the probability of having 3 screens that are − + − or − − + or − + + is 7.9%. For those with an initial negative screen and who have PLCOm2012 risks below the ceiling probability, the subsequent risk of lung cancer is low (Table 2)^b^
Risk prediction model threshold	+ − + +	NA + − +	NA NA NA NA	Offer the next annual screen
Risk prediction model threshold	− − − − + + + +	− − + + − − + +	− + − + − + − +	Base decision to offer the next annual screen on PLCO2012results risk estimate and selected eligibility threshold criteria. Those with any positive screens should be offered annual screens. The PLCO2012results estimates can help prioritize efforts. For example, an individual with PLCOm2012 risk of 3% who ends up with ≥2 positive screens with third positive (group 4) has a PLCO2012results 6-y risk of 15%, which is very high, warranting conscientious monitoring and promotion of annual screening
NLST-like criteria^c^	− − −	NA − −	NA NA −	If enrollment has been based on NLST-like criteria and 1, 2, or 3 negative screens occur, apply the PLCOm2012 model; if 6-y risk is <1.5%, then consider omitting the next screening and reassessing model-based risk on an annual basis for reentry into screening. Reassessment is especially important for those with a new diagnosis of COPD, personal history of cancer, or family history of lung cancer^c^

Abbreviations: COPD, chronic obstructive pulmonary disease; Lung-RADS, Lung CT Screening Reporting & Data System; NA, not available; NLST, National Lung Screening Trial; PLCOm2012, risk prediction model described by Tammemägi et al.^6^

^a^ The minus sign indicates a Lung-RADS score of 1 or 2. The plus sign indicates a Lung-RADS score of 3 or 4, which were not found to be lung cancer.

^b^ At the ceiling probability or above, having 3 consecutive negative screens cannot lower the results-adjusted PLCOm2012 risk score below the eligibility threshold according to estimates prepared by the PLCO2012results model.

^c^ The NLST-like criteria include NLST, US Preventive Services Task Force, and Centers for Medicare & Medicaid Services enrollment criteria, which are based on at least 30 pack-years, quit time less than 15 full years ago, and ages 55 to 80 years for the US Preventive Services Task Force criteria and ages 55 to 77 years for the Centers for Medicare & Medicaid Services criteria. In PLCO smokers who are NLST criteria positive and PLCOm2012 negative (<1.5% risk), the observed 6-year lung cancer risk is 0.8% (95% CI, 0.6%-1.0%), which is generally considered too low to enroll into screening.

The NLST screening results were associated with postscreening lung cancer risk. We modified the PLCOm2012 risk prediction model to incorporate past screening results and predict subsequent 3-year lung cancer risk. The PLCO2012results model was associated with improved overall prediction, discrimination, and the net benefit compared with the analogous model excluding screening results (PLCOm2012).

Patz and colleagues^19^ studied the association of a normal initial screen with incidence of lung cancer in the NLST. In contrast, the present study evaluated the association of all 3 NLST screens with risk (adjusted for other risk factors using a validated risk prediction model), excluded cancers detected during the screening period to develop accurately calibrated risk estimates, removed overdiagnosis bias, and developed a highly predictive risk model incorporating screening results.

The findings by Patz and colleagues^19^ might suggest that high-risk individuals who have a normal initial screen may not need subsequent screening. Our findings show that individuals with a negative initial screen who have 1 or more subsequent positive screens can remain at substantially elevated risk (Table 1). Moreover, some individuals start with such elevated risks that repeated negative screens do not lower their risk estimates below eligibility thresholds. Lung cancer risks in those with initial negative screens are heterogeneous. Overall, 46.1% of individuals with a Lung-RADS T0-negative screen had PLCO2012results 3-year risks exceeding 1.0%, and 183 of 9016 of them (2.0%) were diagnosed as having lung cancer during T3 to T6. Overall, 55.2% (174 of 315) of lung cancers occurring during the follow-up period occurred in individuals who had 3 negative screens (eTable 2 in the Supplement). The decision whether to undertake or omit the next annual screening can be personalized by using a risk model incorporating lung cancer risk factors and screening results over more than 1 screening, when available. If selection of individuals for a biennial screen was properly done based on accurate risk estimation and appropriate low-risk threshold, then there would be minimal or no excess deaths expected (Table 3).

Having 3 consecutive negative screens was associated with lower lung cancer risk below that estimated by PLCOm2012. However, for any given eligibility threshold used to select patients for screening, there is a corresponding ceiling probability of PLCOm2012 risk that is so high that 3 negative screens will not bring the results-adjusted PLCOm2012 score below the screening eligibility threshold. Individuals who have an initial negative screen who have PLCOm2012 scores above the ceiling will remain at high enough risk that they should be offered annual screening. The PLCO2012results calculator can help determine the ceiling for a screening eligibility threshold and thus can help guide decision making after a negative screen. Potential implications of the study findings on screening interval are summarized in Table 3.

Applying the PLCO2012results for planning screening regimens can lead to revision of decisions based on the PLCOm2012. Screening decision based on PLCO2012results estimates should be given priority over the parallel PLCOm2012 estimates because of more accurate predictions.

The PLCO2012results model can also be used for selection of high-risk samples for research purposes, such as evaluation of biomarkers for early detection and prevention trials. The mechanism explaining the association between screening results and increased risk is unknown. One possible explanation is that positive screens are a marker of lung inflammation, which is recognized to be associated with lung carcinogenesis.^35^

### Strengths and Limitations

Strengths of the study include that the findings are based on prospective data. In addition, the results represent outcomes from a large, well-conducted trial (the NLST^2^) that focused on lung cancer screening.

An apparent study limitation of this study might be that lung cancer risks were estimated after 3 screenings, not just 1 or 2. This study found that conclusions drawn after 1 screening are simplifications that do not hold in all circumstances. Moreover, the 20% lung cancer mortality reduction observed in the NLST^2^ was estimated for a 3-screen program, so this seems the natural point at which to evaluate if further screens are warranted accounting for previous screening results.

The findings of the present study need to be validated in prospective, high-quality data in different settings. It needs to be determined if subsequent screen-detected lung cancers that follow previous positive screens are predominantly early stage. The mechanism that leads to increased risk in those with false-positive (non–lung cancer) screens needs explanation. The cost-effectiveness of using PLCO2012results to guide screening decisions requires evaluation.

## Conclusions

The study findings are expected to have clinical and public health implications. Including screening result information was associated with improved lung cancer risk prediction. A primary application of the PLCO2012results model is to help guide the decision when to undertake the next screen once an individual has participated in lung cancer screening. In a cost-effectiveness analysis of lung cancer screening in Ontario, Canada, the primary driver of costs was the computed tomography examinations.^36^ The use of PLCO2012results may lead to fewer screens, which may improve cost-effectiveness and reduce radiation exposure and other screening harms, such as false-positive findings and overdiagnosis.

## Notes

Supplement. **eFigure 1.** Flow Chart Describing NLST Analytic Sample for the Current Study **eFigure 2.** PLCO2012results Model Calibration – Observed vs Predicted 3-Year Lung Cancer Risks From 1 to 4 Years After Last Screen in the LSS (Development) and ACRIN (Validation) LDCT Screening Arms (Line Graphs) **eFigure 3.** Receiver Operator Characteristic Area Under the Curves for Predicting Lung Cancer in the LSS and ACRIN NLST LDCT Arms 1 to 4 Years Post-Screening for the PLCOm2012bu Model and PLCO2012results Model, Which Additionally Includes Lung-RADS Results Coded in Four Groups **eTable 1.** Characteristics of the NLST LDCT Participants Who Were Included in Prediction Modelling Analysis **eTable 2.** Comparison of the Distributions of Dichotomized Lung-RADS Results Over Three Rounds of NLST LDCT Screening in Four Groups and Lung Cancers Occurring 1 to 4 Years Following the Third and Final Screen **eTable 3.** Sensitivity, Specificity, Positive Predictive Value for the PLCOm2012bu and PLCO2012results Models With 3-Year Risk Thresholds of 0.75%, 1.0% and 1.5% in LSS and ACRIN Components of the NLST **eAppendix.** Decision Curve Analysis (DCA) Comparing the Net Benefit of PLCOm2012bu Plus Screening Results (PLCO2012results) vs PLCOm2012bu **eFigure 4.** Decision Curve Analysis Comparing PLCO2012results Which Includes Three Years of Lung-RADS Results Coded Into Four Groups vs the Equivalent Model Excluding Screening Results (PLCOm2012bu†), in NLST LDCT Data (N = 22,229) **eReference**
