# Supplementary material for: Development and Validation of a Multivariable Lung Cancer Risk Prediction Model That Includes Low-Dose Computed Tomography Screening Results: A Secondary Analysis of Data From the National Lung Screening Trial
Source: JAMA Netw Open. 2019 Mar 1;2(3):e190204. doi: 10.1001/jamanetworkopen.2019.0204 (PMC6484623; doi:10.1001/jamanetworkopen.2019.0204)
Supplement: Supplement. — eFigure 1. Flow Chart Describing NLST Analytic Sample for the Current Study eFigure 2. PLCO2012results Model Calibration – Observed vs Predicted 3-Year Lung Cancer Risks From 1 to 4 Years After Last Screen in the LSS (Development) and ACRIN (Validation) LDCT Screening Arms (Line Graphs) eFigure 3. Receiver Operator Characteristic Area Under the Curves for Predicting Lung Cancer in the LSS and ACRIN NLST LDCT Arms 1 to 4 Years Post-Screening for the PLCOm2012bu Model and PLCO2012results Model, Which Additionally Includes Lung-RADS Results Coded in Four Groups eTable 1. Characteristics of the NLST LDCT Participants Who Were Included in Prediction Modelling Analysis eTable 2. Comparison of the Distributions of Dichotomized Lung-RADS Results Over Three Rounds of NLST LDCT Screening in Four Groups and Lung Cancers Occurring 1 to 4 Years Following the Third and Final Screen eTable 3. Sensitivity, Specificity, Positive Predictive Value for the PLCOm2012bu and PLCO2012results Models With 3-Year Risk Thresholds of 0.75%, 1.0% and 1.5% in LSS and ACRIN Components of the NLST eAppendix. Decision Curve Analysis (DCA) Comparing the Net Benefit of PLCOm2012bu Plus Screening Results (PLCO2012results) vs PLCOm2012bu eFigure 4. Decision Curve Analysis Comparing PLCO2012results Which Includes Three Years of Lung-RADS Results Coded Into Four Groups vs the Equivalent Model Excluding Screening Results (PLCOm2012bu†), in NLST LDCT Data (N = 22,229) eReference [file jamanetwopen-2-e190204-s001.pdf]

Supplementary Online Content

Tammemägi MC, ten Haaf K, Toumazis I, et al. Development and validation of a multivariable lung cancer risk prediction model that includes low-dose computed tomography screening results: a secondary analysis of data from the National Lung Screening Trial. *JAMA Netw Open*. 2019;2(3):e190204. doi:10.1001/jamanetworkopen.2019.0204

eFigure 1. Flow Chart Describing NLST Analytic Sample for the Current Study
eFigure 2. PLCO2012results Model Calibration – Observed vs Predicted 3-Year Lung Cancer Risks From 1 to 4 Years After Last Screen in the LSS (Development) and ACRIN (Validation) LDCT Screening Arms (Line Graphs)
eFigure 3. Receiver Operator Characteristic Area Under the Curves for Predicting Lung Cancer in the LSS and ACRIN NLST LDCT Arms 1 to 4 Years Post-Screening for the PLCOm2012bu Model and PLCO2012results Model, Which Additionally Includes Lung-RADS Results Coded in Four Groups
eTable 1. Characteristics of the NLST LDCT Participants Who Were Included in Prediction Modelling Analysis
eTable 2. Comparison of the Distributions of Dichotomized Lung-RADS Results Over Three Rounds of NLST LDCT Screening in Four Groups and Lung Cancers Occurring 1 to 4 Years Following the Third and Final Screen
eTable 3. Sensitivity, Specificity, Positive Predictive Value for the PLCOm2012bu and PLCO2012results Models With 3-Year Risk Thresholds of 0.75%, 1.0% and 1.5% in LSS and ACRIN Components of the NLST
eAppendix. Decision Curve Analysis (DCA) Comparing the Net Benefit of PLCOm2012bu Plus Screening Results (PLCO2012results) vs PLCOm2012bu
eFigure 4. Decision Curve Analysis Comparing PLCO2012results Which Includes Three Years of Lung-RADS Results Coded Into Four Groups vs the Equivalent Model Excluding Screening Results (PLCOm2012bu†), in NLST LDCT Data (N = 22,229)
eReference.

This supplementary material has been provided by the authors to give readers additional information about their work.

eFigure 1. Flow chart describing NLST analytic sample for the current study

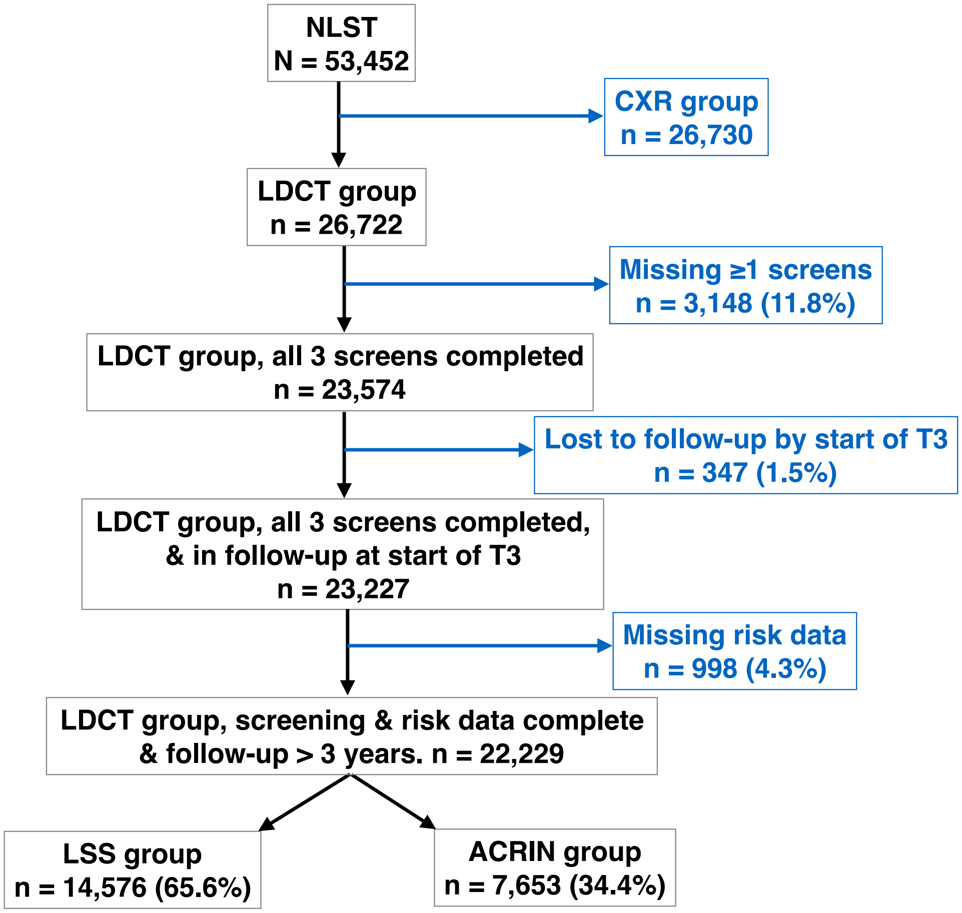

Abbreviations: ACRIN, American College of Radiology Imaging Network; CXR, chest x-ray; LDCT, low dose computed tomography; LSS, Lung Screening Study component of the NLST; N, number in overall sample; n, number in subsample; NLST, National Lung Screening Trial, T3, is the third years after study entry, or one year after the last (T2) screen.

©2019 Tammemägi MC et al. *JAMA Network Open*.

eFigure 2. PLCO2012results* model calibration – observed vs predicted 3-year lung cancer risks from 1 to 4 years after last screen in the LSS (development) and ACRIN (validation) LDCT screening arms (line graphs). Distributions of lung cancer cases and non-cases risks are shown in the scatter plot.

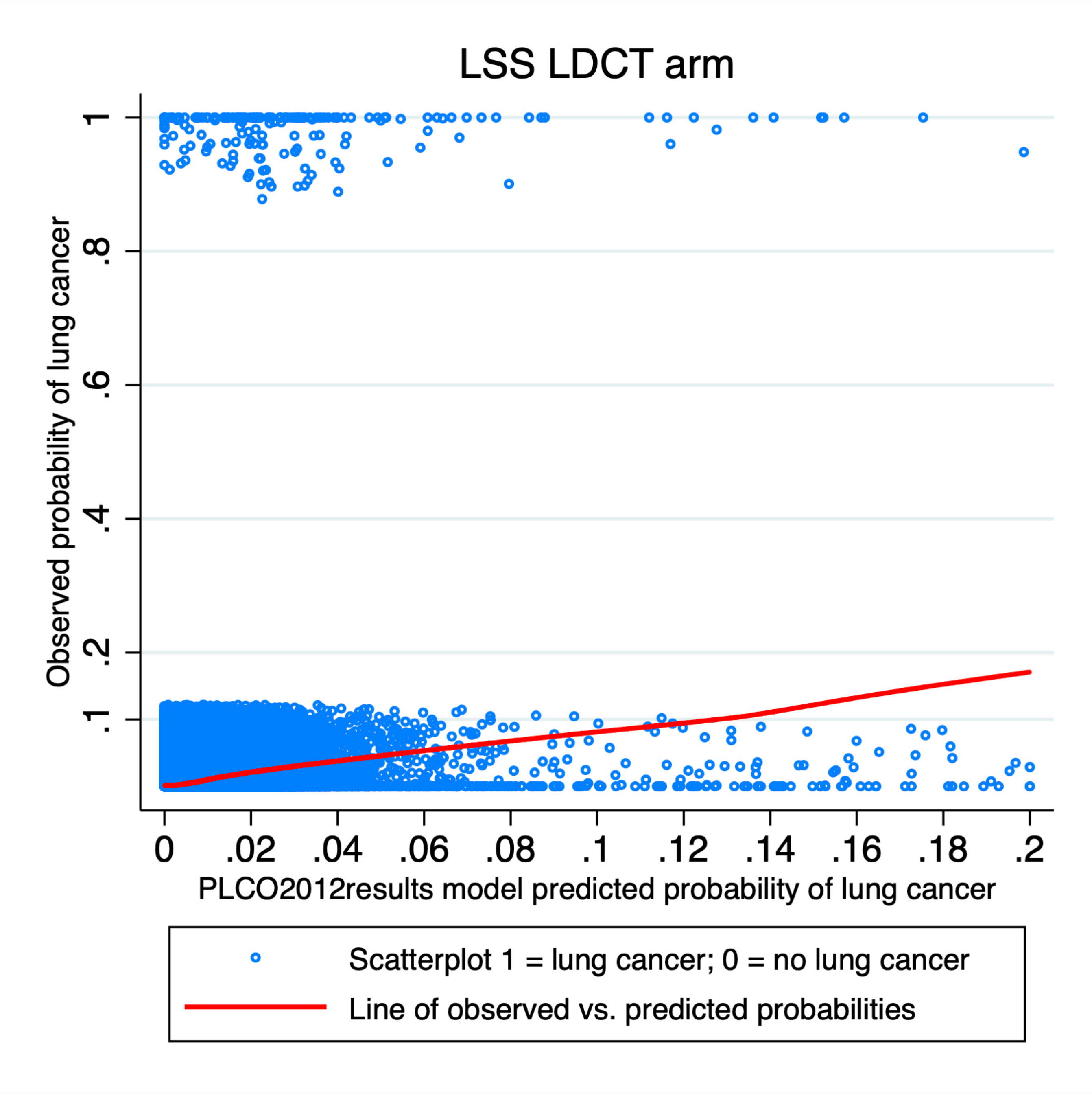

©2019 Tammemägi MC et al. *JAMA Network Open*.

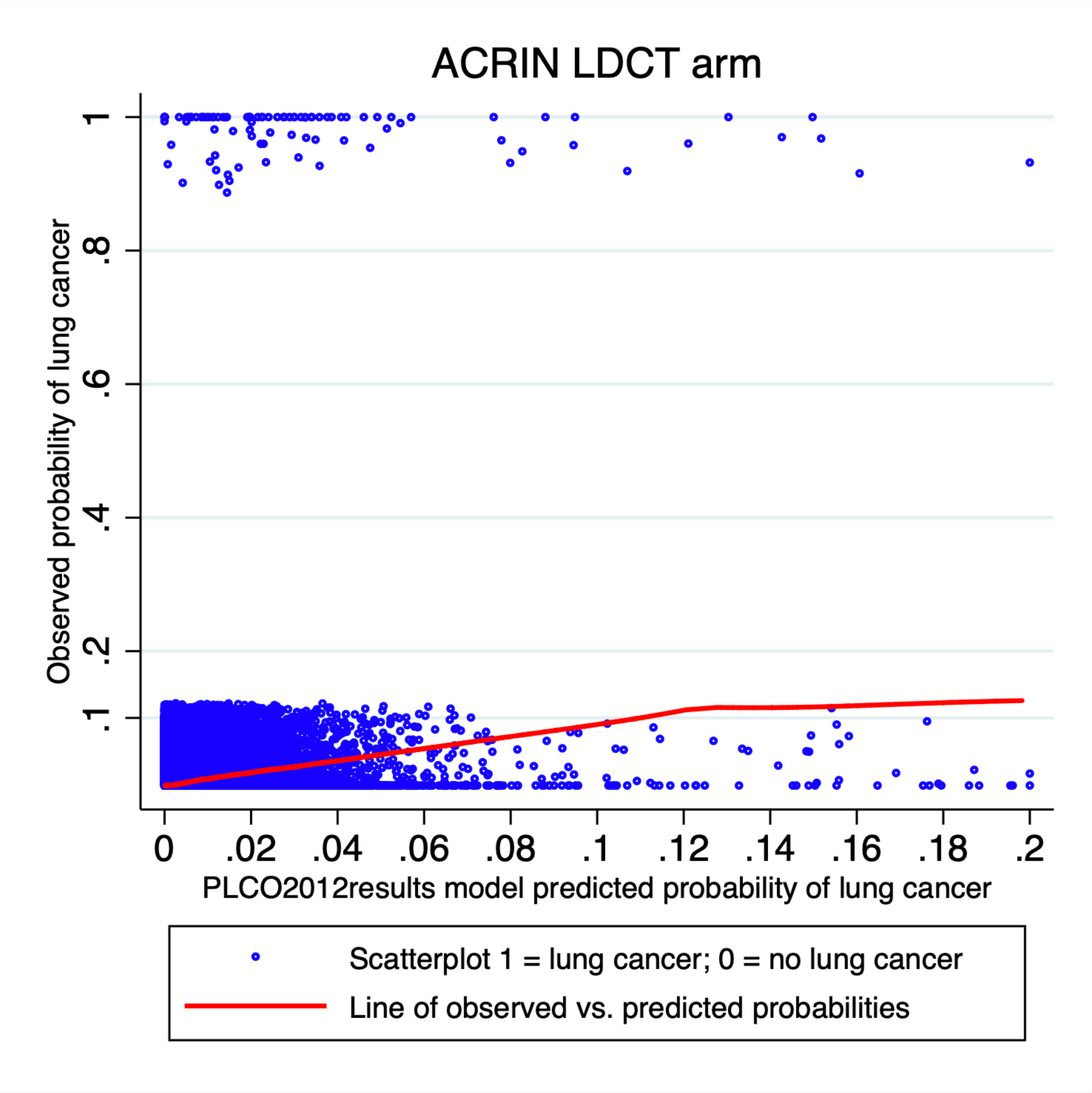

Abbreviations: ACRIN, American College of Radiology Imaging Network subset of the NLST; LDCT, low dose computed tomography; LSS, Lung Screening Study subset of the NLST.

* The PLCO2012results model predicts lung cancer 1 to 4 years after the last screen and includes PLCOm2012$_{bu}$ scores and Lung-RADS results over 3 annual screens in four groupings as predictors. The PLCO2012results model is described in Table 1.

©2019 Tammemägi MC et al. *JAMA Network Open*.

eFigure 3. Receiver operator characteristic area under the curves for predicting lung cancer in the LSS and ACRIN NLST LDCT arms 1 to 4 years post-screening for the PLCOm2012$_{bu}$* model and PLCO2012results† model, which additionally includes Lung-RADS results coded in four groups. P-values for differences in AUCs in LSS and ACRIN are <0.001.

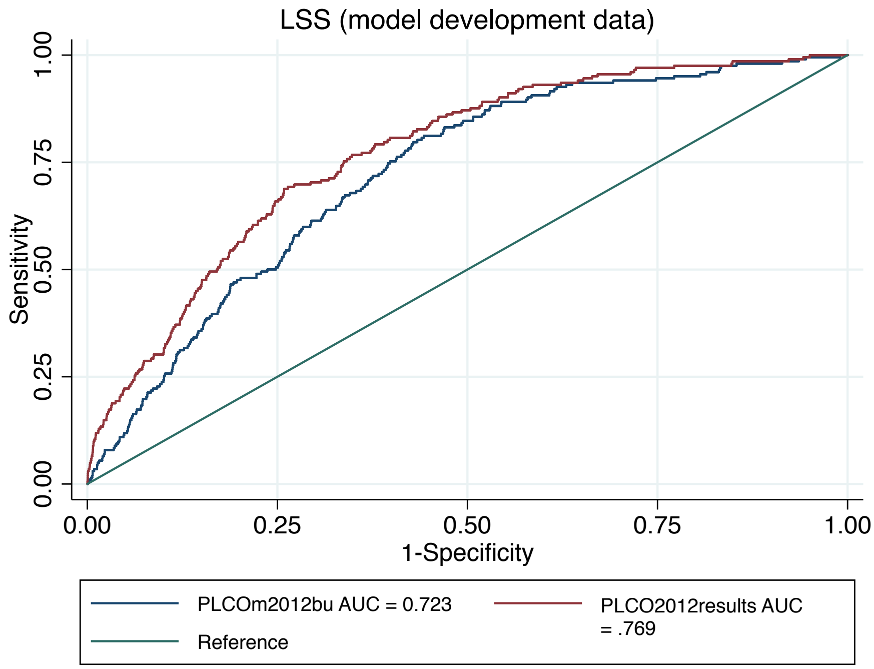

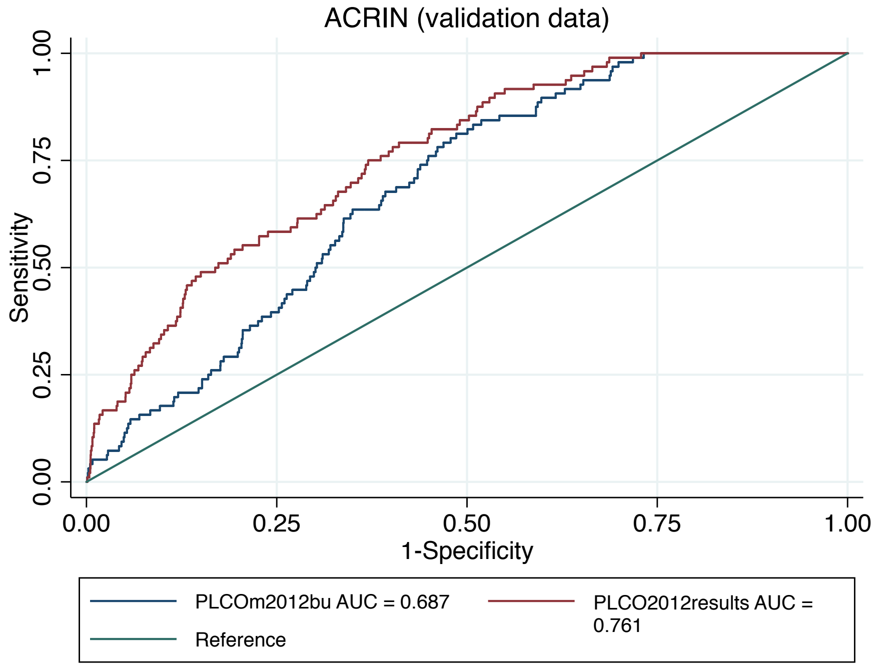

©2019 Tammemägi MC et al. *JAMA Network Open*.

Abbreviations: ACRIN, American College of Radiology Imaging Network subset of the NLST; AUC, receiver operator characteristic area under the curve; LDCT, low dose computed tomography; LSS, Lung Screening Study subset of the NLST; NLST, National Lung Screening Trial; PLCOm2012 is the name of the lung cancer risk prediction model described in reference [1]).

* $PLCOm2012_{bu}$ is the PLCOm2012 model with predictors age, smoking duration in current smokers, and quit-time in former smokers updated to the start of follow-up (T3) by adding 3 years to baseline values, and it is estimated for a three-year period, not the original six-year period, by dividing the estimate by 2.

† The PLCO2012results model predicts lung cancer 1 to 4 years after the last screen and includes $PLCOm2012_{bu}$ scores and Lung-RADS results over 3 rounds in four groupings as predictors. The PLCO2012results model is described in Table 1.

©2019 Tammemägi MC et al. *JAMA Network Open*.

eTable 1. Characteristics of the NLST LDCT participants who were included in prediction modelling analysis

Characteristic	LSS (n = 14576)	ACRIN (n = 7653)	P-value	CT group (n = 22,229)*
Age (years median)	60 (IQR 57-65)	61 (IQR 57-65)	P_{NPT} = 0.005	60 (IQR 57-65
Sex				
Male	8946 (61.4%)	4225 (55.2%)		13171 (59.3%)
Female	5630 (38.6%)	3428 (44.8%)	P_{FET} < 0.001	9058 (40.7%)
Race				
White, not Hispanic	13123 (90.0%)	7087 (92.6%)		20210 (90.9%)
Black, not Hispanic	484 (3.3%)	382 (5.0%)		866 (3.9%)
Hispanic	207 (1.4%)	76 (1.0%)		283 (1.3%)
Asian	420 (2.9%)	48 (0.6)		468 (2.1%)
Native Hawaiian/Pacific Islander	60 (0.4%)	2 (0.0%)		62 (0.3%)
American Indian/Alaskan Native	40 (0.3%)	20 0.3%)		60 (0.3%)
More than one race	242 (1.7%)	38 (0.5%)	P_{CHI} < 0.001	280 (1.3%)
Education				
High school graduate or less	4232 (29.0%)	2210 (28.9%)		6442 (29.0%)
Post-high school education	10344 (71.0%)	5443 (34.5%)	P_{FET} = 0.81	15787 (71.0%)
Body mass index (kg/m^2)	28.0 (SD 4.9)	28.0 (SD 5.0)	P_{TT} = 0.19	28.0 (SD 5.0)
Personal history of cancer				
No	14008 (96.1%)	7349 (96.0%)		21357 (96.1%)
Yes	568 (3.9%)	304 (4.0%)	P_{FET} = 0.80	872 (3.9%)
History of COPD				
No	12309 (84.5%)	6159 (80.5%)		18468 (83.1%)
Yes	2267 (15.5%)	1494 (19.5%)	P_{FET} < 0.001	3761 (16.9%)
Family history of lung cancer				
No	11511 (79.0%)	5834 (76,.2%)		17345 (78.0%)
Yes	3065 (21.0%)	1819 (23.8%)	P_{FET} < 0.001	4884 (22.0%)
Smoking status				
Former smoker	7949 (54.5%)	3891 (50.8%)		11840 (53.3%)
Current smoker	6627 (45.5%)	3762 (49.2%)	P_{FET} < 0.001	10389 (46.7%)
Smoking intensity (cigarettes/day)	28.6 (SD 11.6)	28.1 (SD 11.0)	P_{TT} = 0.001	28.4 (SD 11.4)
Smoking duration (years)	40.5 (SD 7.0)	41.1 (SD 7.1)	P_{TT} < 0.001	40.7 (SD 7.0)
Smoking quit-time in former smokers (years)	10 (n = 7949, IQR 6-14)	10 (n = 3891, IQR 6-14)	P_{NPT} = 0.18	10 (n = 11840, IQR 6-14)

Abbreviations: ACRIN, American College of Radiology Imaging Network subset of the NLST; IQR, interquartile range; LDCT, low dose computed tomography; LSS, Lung Screening Study subset of the NLST; n, subset number; NLST, National Lung Screening Trial; P_{CHI}, p-value based on the likelihood ratio chi-square test; P_{FET}, p-value based on Fisher's exact test; P_{NPT}, p-value based on nonparametric test of trend; P_{TT}, p-value based on the t-test not assuming equal standard deviations; SD, standard deviation.

* This cohort excludes those with missing data: for education for 448 (2.0%), for body mass index for 68 (0.3%), for family history of lung cancer for 294 (1.3%), for race/ethnicity for 85 (0.4%) and for smoking quit-time for 133 (0.6%).

©2019 Tammemägi MC et al. *JAMA Network Open*.

eTable 2. Comparison of the distributions of dichotomized Lung-RADS results* over three rounds of NLST LDCT screening in four groups and lung cancers occurring 1 to 4 years following the third and final screen

4 Groups of Lung-RADS results in 3 rounds of LDCT screening	Lung cancers/n in results strata (% lung cancers in cell) {column %} LSS (n = 15152)	Lung cancers/n in results strata (% lung cancers in cell) {column %} ACRIN (n = 8075)	Lung cancers/n in results strata (% lung cancers in cell) {column %} LSS & ACRIN combined (N = 23227)
Group 1<br>- - -	116/12223 (0.9%)<br>{80.7%}	58/6547 (0.9%)<br>{81.1%}	174/18770 (0.9%)<br>{80.8%}
Group 2<br>+ - - or - + -	43/2028 (2.1%)<br>{13.4%}	19/1020 (1.9%)<br>{12.6%}	62/3048 (2.0%)<br>{13.1%)}
Group 3<br>+ + - or - - +	18/621 (2.9%)<br>{4.1%}	17/353 (4.8%)<br>{4.4%}	35/974 (3.6%)<br>{4.2%}
Group 4<br>+ - + or - + + or + + +	31/280 (11.1%)<br>{1.9%}	13/155 (8.4%)<br>{1.9%}	44/435 (10.1%)<br>{1.9%}

Abbreviations: ACRIN, American College of Radiology Imaging Network subset of the NLST; AUC, LDCT, low dose computed tomography; LSS, Lung Screening Study subset of the NLST; N, number overall; n, subset number; NLST, National Lung Screening Trial.

* Lung-RADS scores of 1 and 2 are negative, and scores of 3 and 4 are positive.

©2019 Tammemägi MC et al. *JAMA Network Open*.

eTable 3. Sensitivity, specificity, positive predictive value for the PLCOm2012$_{bu}$* and PLCO2012results† models with 3-year risk thresholds of 0.75%, 1.0% and 1.5% in LSS and ACRIN components of the NLST

Sample and Model	Risk Threshold = 0.75%			Risk Threshold = 1.0%			Risk Threshold = 1.5%		
	Sensitivity	Specificity	PPV	Sensitivity	Specificity	PPV	Sensitivity	Specificity	PPV
LSS (n = 14,576, LCA=202)									
PLCOm2012$_{bu}$	95.1%	19.6%	1.6%	93.6%	31.6%	1.9%	83.2%	52.5%	2.4%
PLCO2012results	93.1%	41.2%	2.2%	85.6%	53.2%	2.5%	70.3%	70.6%	3.3%
ACRIN (n = 7653, LCA = 96)									
PLCOm2012$_{bu}$	100.0%	16.0%	1.5%	99.0%	27.1%	1.7%	84.4%	47.2%	2.0%
PLCO2012results	93.7%	36.4%	1.8%	85.4%	48.9%	2.1%	65.6%	67.3%	2.5%

Abbreviations: ACRIN, American College of Radiology Imaging Network subset of the NLST**;** LSS, Lung Screening Study subset of the NLST; NLST, National Lung Screening Trial; PPV, positive predictive value.

* PLCOm2012$_{bu}$ is the PLCOm2012 model with predictors age, smoking duration in current smokers, and quit-time in former smokers updated to the start of follow-up (T3) by adding 3 years to baseline values, and it is estimated for a three-year period, not the original six-year period.

† The PLCO2012results model predicts lung cancer 1 to 4 years after the third screen and includes PLCOm2012$_{bu}$ scores and Lung-RADS results over 3 rounds in four groupings as predictors. The PLCO2012results model is described in Table 1.

©2019 Tammemägi MC et al. *JAMA Network Open*.

eAppendix. Decision curve analysis (DCA) comparing the net benefit of $PLCOm2012_{bu}$ plus screening results (PLCO2012results) versus $PLCOm2012_{bu}$

In DCA, to assess the balance between benefits and harms, the number of true positive classifications minus false positive classifications is taken as a proportion of the number of classifications. This ratio is considered at different decision thresholds across a range of risks of interest, and at each threshold, the number of false positives is weighted by the odds of the risk being considered. For example, at a 2% threshold for deciding to screen an individual, the weighting is 0.02/0.98 = 1/49: implying that missing one lung cancer is valued equal to the harm done by 49 false positives.

Decision curve analysis demonstrates that between risk thresholds zero to 6%, the net benefit for model PLCO2012results exceeds that of $PLCOm2012_{bu}$ (eFigure 4). 99.1% of the overall NLST LDCT sample had PLCO2012results 3-year risks ≤6%.

©2019 Tammemägi MC et al. *JAMA Network Open*.

eFigure 4. Decision curve analysis comparing PLCO2012results* which includes three years of Lung-RADS results coded into four groups versus the equivalent model excluding screening results (PLCOm2012$_{bu}$†), in NLST LDCT data (N = 22,22 9)

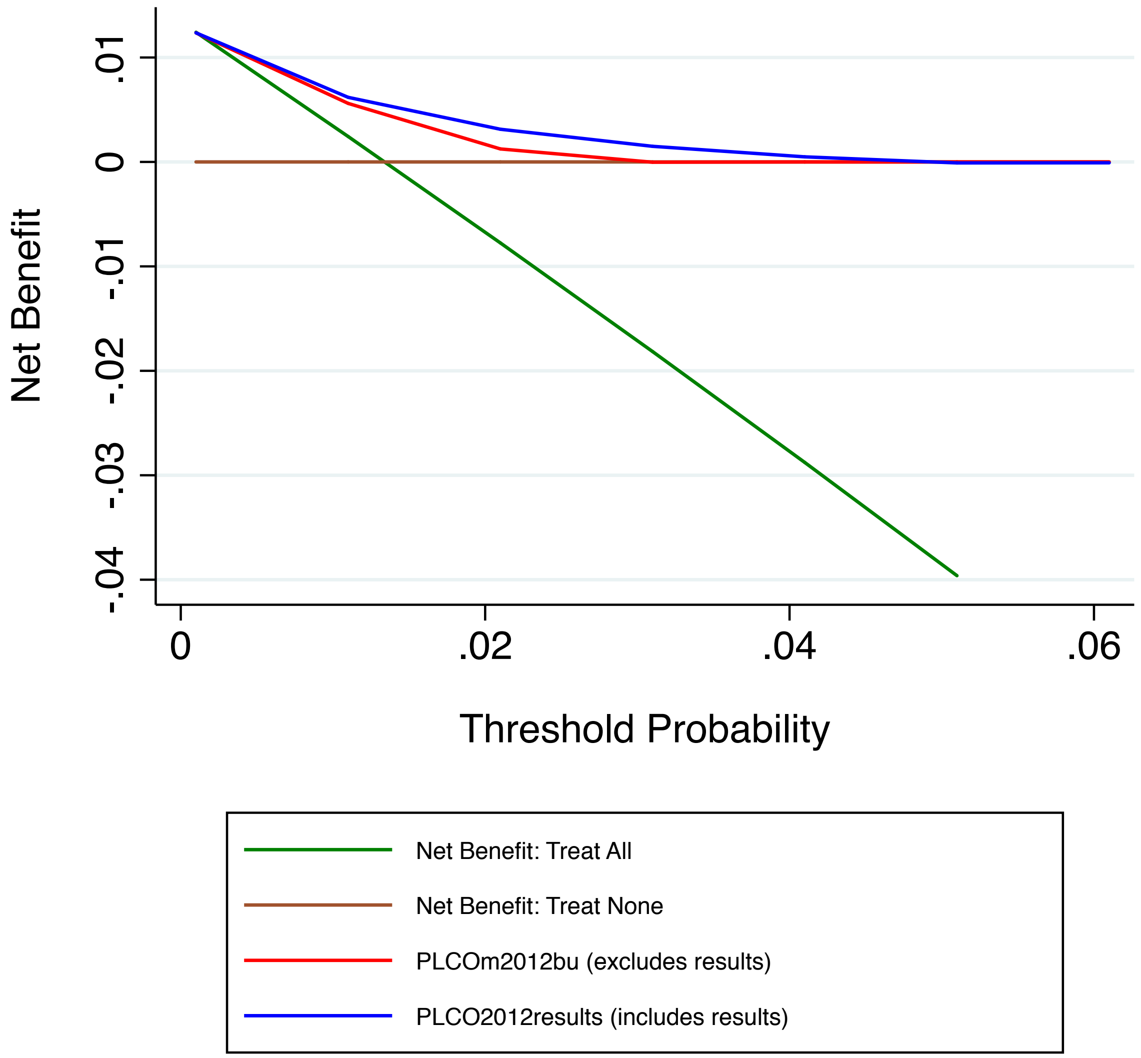

* The PLCO2012results model predicts lung cancer 1 to 4 years after the last screen and includes PLCOm2012$_{bu}$ scores and Lung-RADS results over 3 annual screens in four groupings as predictors. The PLCO2012results model is described in Table 1.

† PLCOm2012$_{bu}$ is the PLCOm2012 model with predictors age, smoking duration in current smokers, and quit-time in former smokers updated to the start of follow-up (T3) by adding 3 years to baseline values, and it is estimated for a three-year period, not the original six-year period used for the PLCOm2012 model[1].

©2019 Tammemägi MC et al. *JAMA Network Open*.

eReference.

1. Tammemagi MC, Katki HA, Hocking WG, et al. Selection criteria for lung-cancer screening. The New England journal of medicine 2013;368:728-36.

©2019 Tammemägi MC et al. *JAMA Network Open*.
